# Synergistic Effect of Fermented Rice Extracts on the Probiotic and Laxative Properties of Yoghurt in Rats with Loperamide-Induced Constipation

**DOI:** 10.1155/2014/878503

**Published:** 2014-08-20

**Authors:** Jae-Suk Choi, Joo Wan Kim, Ki-Young Kim, Jong-Kwang Lee, Jae Hak Sohn, Sae-Kwang Ku

**Affiliations:** ^1^RIS Center, IACF, Silla University, Gwaebup-dong, Sasang-gu, Busan 617-736, Republic of Korea; ^2^Department of Bio-Food Materials, Silla University, Sasang-gu, Busan 617-736, Republic of Korea; ^3^Glucan Corp. No. 305 Marine Bio-Industry Development Center, Hoenggye-ri 27, Ilgwang-myeon, Gijan-gun, Busan 619-912, Republic of Korea; ^4^JKnutra, No. 502, 17 Joongdaero 23-gil, Songpa-ku, Seoul 138-160, Republic of Korea; ^5^Department of Anatomy and Histology, College of Oriental Medicine, Daegu Haany University, 290 Yugok-dong, Gyeongsan-si, Gyeongsanbuk-do 712-715, Republic of Korea

## Abstract

*Aim*. The objective was to evaluate the synergistic effects of fermented rice extracts (FRe) on the laxative and probiotic properties of yoghurt in rats with loperamide-induced constipation. Methods. After constipation induction, yoghurt containing FRe (BFRe; 0.05%, 0.1%, or 1%) was administered orally once per day for 6 days. *Results*. Loperamide treatment caused marked decreases in fecal pellet numbers and water content discharged, as well as in the surface mucosal thickness of the colonic lumen, intestinal charcoal transit ratio, thickness, and number of mucous-producing goblet cells in the colonic mucosa, whereas it increased the remnant fecal pellet number and the mean diameter of the colonic lumen. However, this loperamide-induced constipation was ameliorated by treatment with FRe, yoghurt single formula, or 0.05%, 0.1%, or 1% BFRe (10 mL/kg). Additionally, the viable numbers of * Lactobacillus* in the cecal contents and feces were markedly higher than those in constipated rats. Moreover, greater probiotic and laxative effects were detected in BFRe-treated rats than in rats treated with equivalent doses of yoghurt or FRe single formula. * Conclusion*. The results suggest that addition of FRe to liquid yoghurt will enhance the probiotic and beneficial laxative effects of yoghurt in the digestive tract, without causing side effects.

## 1. Introduction

Constipation is a symptom-based disorder defined as “unsatisfactory defecation, characterized by infrequent stools, difficult stool passage, or both. Difficult stool passage includes straining, a sense of difficulty passing stool, incomplete evacuation, hard/lumpy stools, prolonged time to stool, or need for manual maneuvers to pass stool” [1]. Constipation may be suspected if there is difficulty or pain when passing a hardened stool or if there is more than 3-day lapse between bowel movements [2]. Constipation is common worldwide, affecting all ages, with prevalence of 0.7–29.6% in children and adolescents [3] and 15–50% in the elderly [1]. In the United States, constipation is one of the top five outpatient gastrointestinal diagnoses [4], costing approximately $7,500 (US dollars) for diagnosis and treatment provision [5].

Commonly used pharmacological agents for the treatment of constipation include bulk-forming laxatives (ispaghula husk, methylcellulose, and bran), stimulant laxatives (senna, bisacodyl, sodium picosulfate, and glycerin suppositories), osmotic laxatives (lactulose, magnesium sulphate, and phosphate enema), fecal softeners (docusate sodium, liquid paraffin, and arachis oil), and prokinetic agents (prucalopride and tegaserod) [1, 6]. However, laxatives also may induce side effects, such as severe diarrhea [7]. Thus, many researchers have sought to develop new therapeutic agents for constipation with reduced side effects and improved laxative effects.

Probiotics such as yoghurt-derived* Lactobacillus* have been raised as alternative therapeutics for various digestive disorders, and they also show favorable effects on constipation with fewer toxic effects [8–10]. Probiotics are reportedly effective for chronic constipation in children. Their laxative and abdominal pain-relieving effects are similar to those of magnesium oxide [11]. Additionally, probiotics alleviate colic, one of the symptoms of severe constipation [12].

Loperamide is an agonist of *μ*-opioid receptors. Agonized *μ*-opioid receptors in the intestine inhibit release of endogenous acetylcholine granules [13]. Loperamide-induced delay of colonic transit results in spastic constipation due to the reduction in stool frequency and increased colonic contractions in humans [14]. This drug inhibits intestinal water secretion [15] and colonic peristalsis [16], which extends the fecal evacuation time and delays intestinal luminal transit [17]. Therefore, loperamide-induced constipation is considered to be a model of spastic constipation [18].

Various fermented rice extracts (FRe) have been shown to exert a number of potent pharmacological effects, especially antioxidant [19], anti-inflammatory [20], hypolipidemic [21], neuroprotective [22], antistress, and antifatigue [23] activities, as compared with nonfermented extracts. In addition, favorable pharmacological properties of FRe related to their probiotic effects against enterobacteria, including digestive disorders, have been reported [24–27]. We previously reported on the laxative effects of FRe in normal healthy rats [28] and on loperamide-induced constipation in rats [29]. The laxative and probiotic potentials of yoghurt have been enhanced via modification, such as fermentation and addition of dietary fibers or other probiotics [30–33]. Thus, we considered that the addition of FRe would increase the laxative and probiotic effects of yoghurt.

In the present study, to evaluate the synergistic effects of FRe on the laxative and probiotic properties of yoghurt, changes in fecal parameters, the gastrointestinal transit ratio, fecal mucosal contents, and colonic mucosal histology were monitored, together with* Lactobacillus* numbers in the cecal contents and feces, in loperamide-induced constipated rats after administration of Bulgaris commercial yoghurt containing 0.05%, 0.1%, or 1% FRe (BFRe).

## 2. Materials and Methods

### 2.1. Preparation and Administration of Drugs

FRe (brown powder) used in this study were prepared and supplied by Glucan Corp. (Busan, Korea) according to the methods of Choi et al. [34] and Lee et al. [35]. Bulgaris, a commercial brand of apple-flavored yoghurt, was purchased from Namyang Dairy Products Co. Ltd. (Gongju, Korea). Test agents were administered orally once a day for 6 days. FRe was dissolved in distilled water and administered orally in a volume of 10 mL/kg. All BFRe mixed formulas (0.05%, 0.1%, and 1% FRe in liquid yoghurt) were prepared by direct addition of the appropriate FRe amounts to 10 mL liquid yoghurt. Immediately after mixing, the liquid yoghurts or BFRe were administered orally in a 10 mL/kg volume once per day for 6 continuous days, and, in the constipation control rats, an equal volume of distilled water was administered orally instead of the test agent. In the intact vehicle control, only distilled water or an equal volume of saline was administered orally instead of the test agent or loperamide, respectively.

### 2.2. Animals and Grouping

A total of 40 specific-pathogen-free male Sprague-Dawley rats (6 weeks old upon receipt; SLC, Japan) were used after acclimatization for 7 days. Five animals were allocated per polycarbonate cage in a room with controlled temperature (20–25°C) and humidity (50–55%). The rats were kept under a 12 h : 12 h light: dark cycle, and food (Samyang, Korea) and water were supplied* ad libitum*. All animals were fasted overnight (~18 h) after the first and last administration of food (water was not restricted). Five rats each were allocated to the following groups (eight groups total): vehicle control (saline or distilled water administered), constipation (loperamide) control (3 mg/kg loperamide + distilled water), FRe 5 group (3 mg/kg loperamide + 5 mg/kg FRe), FRe 10 group (3 mg/kg loperamide + 10 mg/kg FRe), liquid yoghurt group (3 mg/kg loperamide + 10 mL/kg Bulgaris), BFRe 0.05% group (3 mg/kg loperamide + Bulgaris containing 0.05% FRe), BFRe 0.1% group (3 mg/kg loperamide + Bulgaris containing 0.1% FRe), and BFRe 1% group (3 mg/kg loperamide + Bulgaris containing 1% FRe). All animals were treated in accordance with the Guidelines for Care and Use of Laboratory Animals of Daegu Haany University, Gyeongsan, Gyeongbuk, Republic of Korea.

### 2.3. Induction of Constipation in Rats

Constipation was induced in the animals by oral administration of 3 mg/kg loperamide hydrochloride (Sigma, MO, USA) in a volume of 5 mL/kg (dissolved in saline), once per day for 6 continuous days 1 h before administration of the test agent, as described previously [36, 37]; the control rats were administered normal saline only.

### 2.4. Enumeration of* Lactobacillus*

After sacrifice of the rats, viable* Lactobacillus* in the cecal contents and feces were enumerated using the candle jar method (plate-in-bottle method) [38, 39] with Difco Lactobacilli MRS agar (Becton, Dickinson and Company, MD, USA). Briefly,* Lactobacillus* selective agar plates containing 10-fold dilutions of the test agents were incubated at 37°C for 96 h under anaerobic conditions using candle jars. After 96 h of incubation, colonies were counted (×10^8^ colony-forming units (CFU)/mL). In addition,* Lactobacillus* numbers were determined (×10^8^ CFU/mg) after sacrifice of the rats.

### 2.5. Changes in Body Weight

The body weight of each rat was measured using an automatic electronic balance (Precisa Instrument Ag, Switzerland) once per day beginning 1 day before test agent administration until termination. At termination, all experimental animals were fasted overnight (~12 h, with water provided), to reduce the differences attributed to feeding, for measurement of the intestinal charcoal transit ratio. Body weight gains were also measured.

### 2.6. Fecal Parameter Measurement

The excreted fecal pellets of individual rats were collected from their metabolic cages (Harvard Apparatus Ltd., Edenbridge, Kent, UK) 24 h after the fourth administration of test agents. The total number, wet weight, and water content of the fecal pellets were determined. The water content was calculated as the difference between the wet and dry weights of the pellet.

### 2.7. Measurement of the Intestinal Charcoal Transit Ratio

The assessment of gastrointestinal propulsion of the charcoal meal was determined according to Sagar et al. [40] with minor modifications. Test animals were starved for 18 h prior to the experiment but were allowed access to water* ad libitum*. Ten minutes after the sixth (final) administration of the test agent, animals from each group were fed 1 mL charcoal meal (3% activated charcoal suspension in 0.5% aqueous methylcellulose (Sigma, MO, USA)). Thirty minutes after administration of the charcoal meal, the animals from each group were euthanized by cervical dislocation, and the total intestinal length (pyloric sphincter to caecum), as well as the charcoal transit distance as a fraction of that length, was measured. The intestinal charcoal transit ratio was calculated as the difference between the total small intestinal length and the charcoal meal transit distance as follows: charcoal transit ratio (%) = ((total small intestinal length − charcoal meal transit distance)/total small intestinal length) × 100.

### 2.8. Measurement of Fecal Pellets in the Large Intestine

At the time of intestinal charcoal transit ratio measurement, the total numbers and mean thicknesses (short axis) of remnant fecal pellets in the colonic lumen were determined.

### 2.9. Histological Procedures

Histological assessment of the colonic mucosa and remnant fecal pellets in the colonic lumen was performed according to the method of Wu et al. [41], with minor modifications. Briefly, segments of the rat distal colon containing one fecal pellet were isolated using ligatures, removed, and immediately fixed with 10% formaldehyde at the time of intestinal charcoal transit ratio measurement. The fixed tissue segments were embedded in paraffin and serially cut into 3 *μ*m-thick cross sections. The sections were stained with Alcian Blue at pH 2.5. Five tissue segments per group were prepared and the histological profiles interpreted. The histopathologist was blinded to the group distributions at the time of analysis. The mean thickness of the mucosal layers at the fecal surface (*μ*m/fecal pellet), mucous-producing cell numbers (cells/mm^2^ of colonic mucosa), and the colonic mucosal thickness (*μ*m/colon) were measured by histomorphometry using an automated image analyzer (DMI-300, DMI, Korea) under a light microscope.

### 2.10. Statistical Analyses

Multiple comparison tests of the treatment groups were conducted. Variance homogeneity was examined using the Levene test. If the Levene test indicated no significant deviations from variance homogeneity, the obtained data were subjected to one-way ANOVA, followed by the least significant differences (LSD) multiple comparisons test to determine which pairs differed significantly. In cases of significant deviations from variance homogeneity according to the Levene test, the nonparametric Kruskal-Wallis* H* test was conducted. When a significant difference was identified by the Kruskal-Wallis* H* test, the Mann-Whitney* U* (MW) test was used to determine the significance of specific pairwise comparisons. Statistical analyses were conducted using SPSS for Windows (release 14K, SPSS Inc., USA). In addition, the percentage change between the vehicle and loperamide controls was calculated to indicate the severity of induced constipation and then compared with the loperamide control to evaluate the efficacy of the test agents. Significance was indicated by *P* < 0.05 or *P* < 0.01.

## 3. Results

### 3.1. Effects on Body Weight

No meaningful changes in body weight gain were detected in the rats after administration of any of the test agents compared with the intact vehicle or loperamide control (Table 1).

### 3.2. Effects on Fecal Parameters

Significant (*P* < 0.01) decreases in fecal number and water content collected 24 h after treatment were detected in the loperamide control compared with the vehicle control. However, significant (*P* < 0.01 or *P* < 0.05) increases in fecal number, wet weight, and water content were detected 4 days after administration of 5 or 10 mg/kg FRe, the liquid yoghurt single formula, or all three BFRe concentrations, while marked increases in fecal dry weight were observed in all rats, compared with the loperamide control. More favorable effects on fecal parameters were detected with all three concentrations of BFRe, compared with the equivalent doses of liquid yoghurt or FRe single formula. Therefore, increases in fecal parameters in the treatment groups were in the order BFRe 1% > BFRe 0.1% >> BFRe 0.05% > liquid yoghurt single formula > FRe 10 >> FRe 5, as compared with the loperamide control (Table 2).

The fecal numbers changed by −63.41% in the loperamide control and by 86.67, 106.67, 123.33, 130.00, 166.67, and 180.00% in the FRe 5 and 10, liquid yoghurt single formula, and BFRe 0.05, 0.1, and 1% groups, respectively, as compared with the loperamide control. The fecal wet weights changed by −70.90% in the loperamide control and by 93.85, 103.45, 120.43, 126.19, 141.94, and 165.53% in the FRe 5 and 10, liquid yoghurt single formula, and BFRe 0.05, 0.1, and 1% groups, respectively, as compared with the loperamide control. The fecal dry weights changed by −37.89% in the loperamide control and by 50.09, 39.36, 40.65, 34.51, 34.17, and 33.24% in the FRe 5 and 10, liquid yoghurt single formula, and BFRe 0.05, 0.1, and 1% groups, respectively, as compared with the loperamide control. The fecal water contents changed by −76.04% in the loperamide control and by 124.76, 185.64, 216.65, 240.15, 263.88, and 291.26% in the FRe 5 and 10, liquid yoghurt single formula, and BFRe 0.05, 0.1, and 1% groups, respectively, as compared with the loperamide control.

### 3.3. Effects on Remnant Fecal Pellets in the Lumen of the Colon

Significant (*P* < 0.01) increases in the numbers and mean diameters of fecal pellets remaining in the colonic lumen were detected in the loperamide control compared with the vehicle control, respectively. Significant (*P* < 0.01) decreases in remnant fecal numbers in the colonic lumen at the time of sacrifice after 18 h of fasting were detected in all treated rats compared with the loperamide control. The mean diameters of the remnant fecal pellets were also significantly (*P* < 0.01) decreased in treated rats compared with the loperamide control, with the exception of the FRe 5 group, which showed a nonsignificant decrease. More favorable effects on the remnant fecal pellets in the colonic lumen were detected with all three BFRe concentrations compared with equivalent doses of liquid yoghurt or FRe single formula. Therefore, decreases in the numbers and diameters of remnant fecal pellets in the colonic lumen at the time of sacrifice were in the treatment group order BFRe 1% > BFRe 0.1% >> BFRe 0.05% > liquid yoghurt single formula > FRe 10 > FRe 5, as compared with the loperamide control (Table 3).

The total remnant fecal numbers changed by 84.21% in the loperamide control and by −37.24, −42.86, −45.71, −65.71, −77.14, and −82.86% in the FRe 5 and 10, liquid yoghurt single formula, and BFRe 0.05, 0.1, and 1% groups, respectively, as compared with the loperamide control. The mean diameters of remnant fecal pellets changed by 84.47% in the loperamide control and by −12.44, −20.58, −28.15, −33.06, −42.21, and −56.18% in the FRe 5 and 10, liquid yoghurt single formula, and BFRe 0.05, 0.1, and 1% groups, respectively, as compared with the loperamide control.

### 3.4. Effects on Intestinal Charcoal Transit

A significant (*P* < 0.01) decrease in the intestinal charcoal transit ratio was detected in the loperamide control compared with the vehicle control. Dramatic increases in the intestinal charcoal transit ratio were detected after 6 days of continuous oral treatment with 5 or 10 mg/kg FRe, liquid yoghurt single formula, or the three BFRe concentrations, as compared with the loperamide control. More favorable increases in intestinal charcoal transit were detected in all three BFRe concentration groups compared with the equivalent liquid yoghurt dose or FRe single formula groups. Therefore, increases in the intestinal charcoal transit ratio were detected in the treatment group order BFRe 1% > BFRe 0.1% >> BFRe 0.05% > liquid yoghurt single formula > FRe 10 > FRe 5 groups, as compared with the loperamide control (Table 4).

The intestinal charcoal transit ratio changed by −31.10% in the loperamide control and by 17.54, 20.56, 25.99, 27.82, 58.91, and 73.57% in the FRe 5 and 10, liquid yoghurt single formula, and BFRe 0.05, 0.1, and 1% groups, respectively, as compared with the loperamide control.

### 3.5. Effects on* Lactobacillus* Numbers in the Cecal Contents and Feces

**Figure 1:**
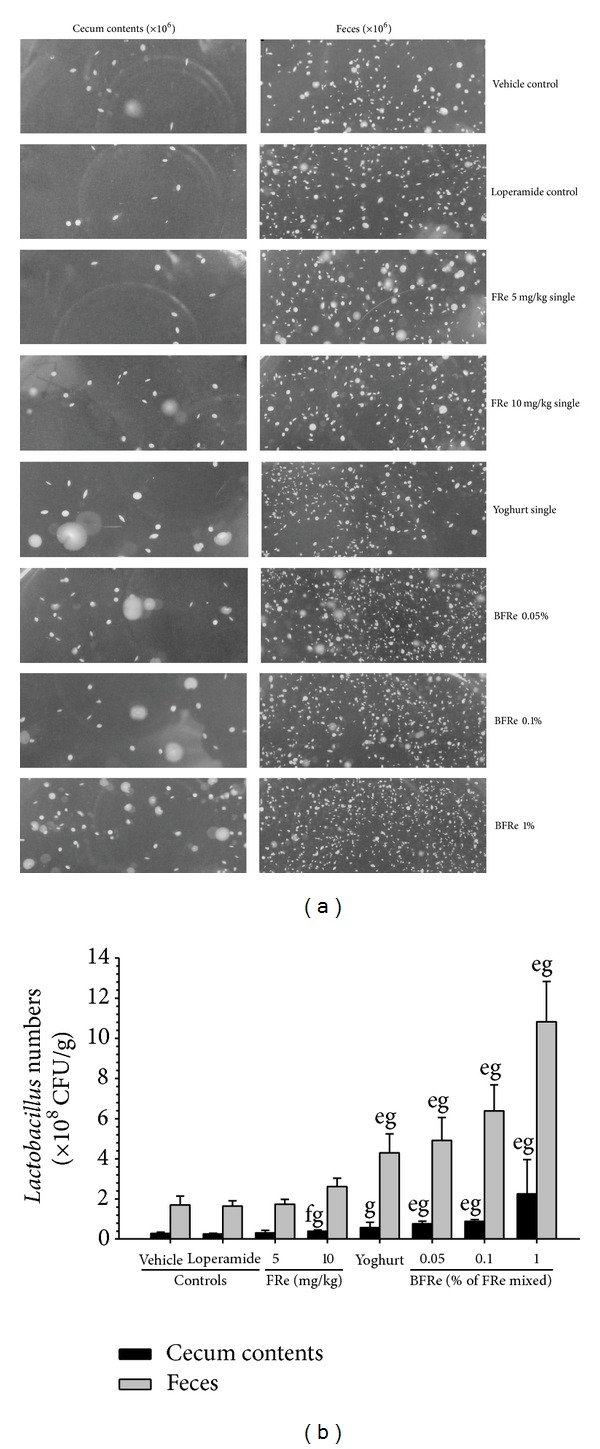
((a), (b)).* Lactobacillus* numbers in the cecal contents and feces of loperamide-induced constipated rats after oral treatment of the test agents. Significant increases in viable* Lactobacillus* numbers were detected in cecal contents and feces 6 days after continuous oral treatment of 10 mg/kg FRe, liquid yoghurt single formula, or the three concentrations of BFRe, as compared with the loperamide control. In addition, greater increases in* Lactobacillus* numbers were detected with all three BFRe concentrations compared with equivalent doses of liquid yoghurt or FRe single formula. Values are expressed as means ± SD of five independent culture plates. The test agents, diluted 10-fold, were incubated at 37°C for 96 h under anaerobic conditions using candle jars, and all were administered in a 10 mL/kg volume. All solutions were administered as 10 mL/kg doses. ^a^ *P* < 0.01 and ^b^ *P* < 0.05 compared with the vehicle control by the LSD test. ^c^ *P* < 0.01 and ^d^ *P* < 0.05 compared with the loperamide control by the LSD test. ^e^ *P* < 0.01 and ^f^ *P* < 0.05 compared with the vehicle control by the MW test. ^g^ *P* < 0.01 and ^h^ *P* < 0.05 compared with the loperamide control by the MW test.

Significant (*P* < 0.01) increases in viable* Lactobacillus* numbers were detected in the cecal contents and feces 6 days after continuous oral treatment with 10 mg/kg FRe, liquid yoghurt single formula, or all three BFRe concentrations, compared with the loperamide control. In addition, greater increases in* Lactobacillus* numbers were detected in all three BFRe concentration groups compared with the groups treated with equivalent doses of liquid yoghurt or FRe single formula. The increases in viable* Lactobacillus* numbers in cecal contents and feces were in the treatment group order BFRe 1% >> BFRe 0.1% > BFRe 0.05% > liquid yoghurt single formula >> FRe 10 >>> FRe 5, compared with the loperamide control (Figures 1(a), and 1(b)).

The viable* Lactobacillus* numbers in the cecal contents were changed by −8.97% in the loperamide control and by 19.70, 51.52, 120.45, 193.18, 237.12, and 754.55% in the FRe 5 and 10, liquid yoghurt single formula, and BFRe 0.05, 0.1, and 1% groups, respectively, as compared with the loperamide control. The viable* Lactobacillus* numbers in the feces of the loperamide control changed by −3.40%, and those in the FRe 5 and 10, liquid yoghurt single formula, and BFRe 0.05, 0.1, and 1% groups changed by 5.95, 59.05, 161.00, 198.78, 288.21, and 557.96%, respectively, as compared with the loperamide control.

### 3.6. Effects on Histopathology

**Figure 2:**
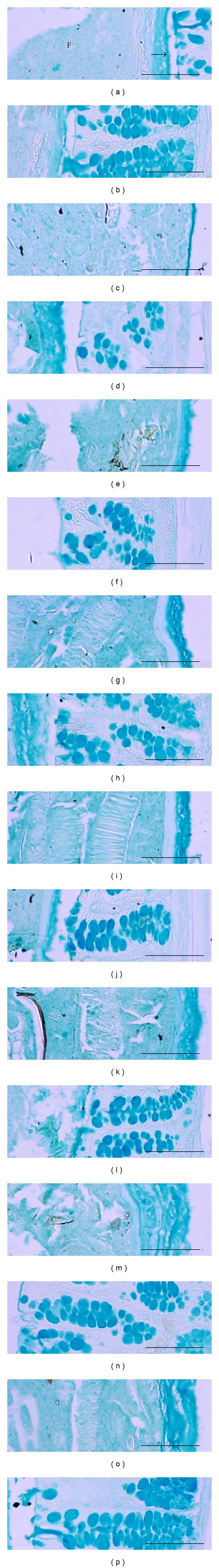
Histological profiles of the fecal pellet-containing colons of loperamide-induced constipated rats treated with the vehicle control ((a), (b)), loperamide control ((c), (d)), 5 mg/kg FRe ((e), (f)), 10 mg/kg FRe ((g), (h)), yoghurt ((i), (j)), and BFRe 0.05% ((k), (l)), 0.1% ((m), (n)), and 1% ((o), (p)) (10 mL/kg). Note the marked decreases in the surface mucosal thickness of remnant fecal pellets in the colonic lumen, mucosal thickness. And mucous-producing cell numbers were detected in the loperamide control, as compared with the vehicle control. However, dramatic increases in these same parameters were detected after 6 days of continuous oral treatment of all test agents, as compared with the loperamide control. In addition, more favorable histopathological changes in the colonic mucosa and the surface mucous of remnant fecal pellets in the colon were detected with all three BFRe concentrations compared with equivalent doses of liquid yoghurt or FRe single formula. Yoghurt: Bulgaris, a brand of commercial yoghurt (Namyang, Korea). FRe: fermented rice extracts. BFRe: mixed formula consisting of yoghurt and the appropriate percentages (0.05, 0.1, and 1%) of FRe. Values are expressed mean ± SD of five rats. The arrow indicates the measured surface mucosal thickness. M, colonic mucosa; F, fecal pellets. All stained with Alcian Blue. Scale bars = 150 *μ*m.

Significant (*P* < 0.01) decreases in the surface mucosal thickness of remnant fecal pellets in the colonic lumen, the mucosal thickness, and mucous-producing cell numbers were detected in the loperamide control compared with the vehicle control. However, significant (*P* < 0.01) increases in these same parameters, compared with the loperamide control, were detected 6 days after continuous oral treatment with all test materials evaluated, with the exception of 5 mg/kg FRe, which showed nonsignificant increases in the surface mucosal thickness of the remnant fecal pellets. In addition, all three concentrations of BFRe, as compared with equivalent doses of liquid yoghurt or FRe single formula, caused more favorable histopathological changes in the colonic mucosa and surface mucous of remnant fecal pellets in the colon. These beneficial effects on histopathological profiles were in the treatment group order BFRe 1% > BFRe 0.1% > BFRe 0.05% > liquid yoghurt single formula > FRe 10 >> FRe 5, as compared with the loperamide control (Table 5; Figure 2).

The surface mucosal thickness of remnant fecal pellets in the colonic lumen of the loperamide control changed by −67.48%, and those of the FRe 5 and 10, liquid yoghurt single formula, and BFRe 0.05, 0.1, and 1% groups changed by 49.23, 168.08, 214.30, 244.96, 482.88, and 616.28%, respectively, as compared with the loperamide control. The number of mucous-producing cells in the colonic mucosa changed by −77.62% in the loperamide control and by 97.75, 162.07, 216.84, 233.02, 265.92, and 296.42% in the FRe 5 and 10, liquid yoghurt single formula, and BFRe 0.05, 0.1, and 1% groups, respectively, as compared with the loperamide control. The thickness of the colonic mucosa changed by −43.27% in the loperamide control and by 16.58, 36.42, 37.75, 53.39, 64.58, and 80.06% in the FRe 5 and 10, liquid yoghurt single formula, and BFRe 0.05, 0.1 and 1% groups, respectively, as compared with the loperamide control.

## 4. Discussion

### 4.1. Laxative Effect of FRe on Constipation in Rats

Constipation is a common public health problem with a well-recognized propensity to cause discomfort and to affect quality of life [7, 37]. Constipation increases during aging and can be a chronic condition requiring the long-term use of laxatives and arising from a variety of causes, including chemical compounds such as morphine, dietary habits, and psychological stress [7]. Loperamide-induced constipation is considered to be a model of spastic constipation [18]. Various FRe exhibit enhanced bioavailabilities and pharmacological activities [19–23], and the laxative and probiotic potentials of yoghurt have been increased by modifications such as fermentation and addition of dietary fibers or other probiotics [30–33]. Thus, we considered that the addition of FRe would increase the laxative and probiotic effects of yoghurt. Therefore, in the present study, the synergistic effects of FRe on the laxative and probiotic properties of yoghurt were evaluated in loperamide-induced constipated rats.

In constipation, marked decreases in fecal discharge and delayed fecal pellet transit in the large intestinal lumen caused by absorption of water into the fecal pellets are observed; accordingly, the water content of the discharged fecal pellets is decreased markedly. Therefore, these changes in fecal parameters, including discharged fecal pellet number and water content, have been used as indices of the effects of various laxative agents [37, 41]. The increases in discharged fecal pellet number and water content in the constipated rats induced by treatment with either FRe dosage, the liquid yoghurt single formula, or the three BFRe concentrations were considered direct evidence of the beneficial laxative effects of these agents. The enhanced laxative effects of yoghurt after the addition of FRe provided further direct evidence, as more favorable changes in fecal parameters, especially water content, occurred after the 0.05, 0.1, and 1% BFRe treatments compared with equivalent doses of liquid yoghurt or FRe single formula. In addition, the increases in remnant fecal pellet numbers in the colonic lumen and decreases in their surface mucosal contents have been associated with constipation [41, 42]. Therefore, the increased fecal surface mucosal thickness and decreased numbers and mucosal thicknesses of remnant fecal pellets in the colonic lumen after treatment with FRe, liquid yoghurt single formula, or all three BFRe concentrations represent further direct evidence of their laxative effects. More favorable effects on remnant fecal pellets in the colonic lumen were detected with all three BFRe concentrations compared with equivalent doses of liquid yoghurt or FRe single formula, indicating that the appropriate addition of FRe to liquid yoghurt enhances the laxative effects and ameliorates constipation.

Transit through the gastrointestinal tract reflects the overall gastrointestinal motor activity, and measurement of the gastrointestinal charcoal transit ratio is useful for the diagnosis of constipation [37]. A decrease in the gastrointestinal charcoal transit ratio is indicative of constipation [40, 43]. Therefore, the increases in gastrointestinal charcoal transit ratio induced by treatment with all test agents were indirect evidence of the beneficial laxative effects of the agents. Moreover, marked increases in the charcoal transit ratio were detected with 0.05, 0.1, and 1% BFRe treatments compared with equivalent doses of liquid yoghurt or FRe single formula. These findings are in agreement with those regarding the fecal parameters, fecal surface mucosal thickness, and* Lactobacillus* numbers in this study.

Reduced mucous production in the colonic mucosa is directly related to constipation [42], and marked decreases in the colonic mucosal layer thickness and mucous-producing cell numbers were detected by histopathology in the constipated rats [44]. Therefore, the increases in mucous-producing cell numbers and mucosal thicknesses were direct evidence of the laxative effects of FRe, liquid yoghurt single formula, and BFRe. In the present study, 0.05, 0.1, and 1% BFRe caused greater increases in mucous-producing cell numbers and mucosal thicknesses in loperamide-induced constipated rats, as compared with equivalent doses of liquid yoghurt or FRe single formula. This suggests that appropriate addition of FRe to liquid yoghurt enhances the laxative effects and will ameliorate constipation.

The intestinal flora plays an important role in the physiological functions of the alimentary tract, and probiotics—such as yoghurt-derived* Lactobacillus*—have been shown to exert favorable effects on various digestive tract disorders, such as inflammatory bowel syndrome and constipation, with lower toxicity [8–10]. Therefore, appropriate addition of FRe to liquid yoghurt is considered to enhance the probiotic effects of yoghurt, because greater numbers of viable* Lactobacillus* spp. were detected in the cecal contents and feces of the BFRe 0.05, 0.1, and 1% groups, compared with single liquid yoghurt or FRe single formula-treated rats.

When FRe were orally administered in this study, we noted a decrease in the numbers of fecal pellets in the colon with an increase in gastrointestinal motility as well as an increase in the numbers of* Lactobacillus* within the fecal pellets. The mechanism of action of FRe seems to be similar to that of prokinetic agents, and an additional mechanism likely involves* Lactobacillus*-growth-stimulating effect as a prebiotic. Abnormal intestinal conditions are improved by both of these mechanisms.

### 4.2. Conclusion

The results of this study suggest that addition of FRe to liquid yoghurt enhances the probiotic and beneficial laxative effects of yoghurt in the digestive tract, without causing side effects. This was supported by the more favorable probiotic and laxative effects of the 0.05, 0.1, and 1% BFRe treatments compared with equivalent doses of liquid yoghurt or FRe single formula in loperamide-induced constipated rats. Therefore, appropriate compositions of BFRe may be an effective complementary treatment for certain types of constipation.

## Figures and Tables

**Table 1:** Body weight gain in loperamide-induced constipated rats during oral treatment of the test agents.

Groups	Body weights at			Body weight gains (B − A)
	Initiation of treatment (A)∗	5th treatment day	Last 6th treatment day (B)∗	
Controls				
Vehicle	208.80 ± 4.97	263.00 ± 9.06	234.80 ± 9.12	26.00 ± 4.53
Loperamide	207.40 ± 3.97	258.20 ± 4.09	232.20 ± 5.76	24.80 ± 3.42
Single formula				
FRe 5 mg/kg	209.60 ± 6.31	257.60 ± 7.50	233.20 ± 8.44	23.60 ± 3.65
FRe 10 mg/kg	209.00 ± 5.48	260.00 ± 12.06	233.60 ± 9.81	24.60 ± 4.67
Yoghurt	208.80 ± 8.23	259.40 ± 11.87	234.60 ± 11.72	25.80 ± 3.90
Mixed formula				
BFRe 0.05%	209.00 ± 7.52	256.00 ± 8.51	232.00 ± 9.14	23.00 ± 2.55
BFRe 0.1%	211.40 ± 4.88	262.40 ± 7.23	236.20 ± 5.81	24.80 ± 1.10
BFRe 1%	209.60 ± 3.65	258.80 ± 0.84	235.40 ± 2.79	25.80 ± 1.48

Values are expressed as means ± SD of five rats, g.

Yoghurt: Bulgaris, a brand of commercial yoghurt (Namyang, Korea).

FRe: fermented rice extracts.

BFRe: mixed formula consisting of yoghurt and the appropriate percentages (0.05, 0.1, and 1%) of FRe.

All solutions were administered as 10 mL/kg doses.

∗Fasted overnight.

**Table 2:** Fecal parameters in loperamide-induced constipated rats after oral treatment of the test agents.

Groups	Fecal Parameters			
	Numbers	Wet weights (g)	Dry weights (g)	Water contents (%)
Controls				
Vehicle	16.40 ± 2.07	3.07 ± 0.88	1.24 ± 0.37	58.82 ± 7.79
Loperamide	6.00 ± 2.00^a^	0.89 ± 0.37^e^	0.77 ± 0.32^a^	14.09 ± 3.10^a^
Single formula				
FRe 5 mg/kg	11.20 ± 0.84^ac^	1.73 ± 0.58^fh^	1.16 ± 0.30^d^	31.68 ± 8.49^ac^
FRe 10 mg/kg	12.40 ± 1.14^ac^	1.82 ± 0.37^fg^	1.08 ± 0.19	40.26 ± 6.49^ac^
Yoghurt	13.40 ± 2.97^bc^	1.97 ± 0.37^fg^	1.09 ± 0.20	44.63 ± 4.12^ac^
Mixed formula				
BFRe 0.05%	13.80 ± 1.92^bc^	2.02 ± 0.34^g^	1.04 ± 0.09	47.94 ± 6.01^bc^
BFRe 0.1%	16.00 ± 1.22^c^	2.16 ± 0.61^g^	1.04 ± 0.30	51.28 ± 8.82^c^
BFRe 1%	16.80 ± 2.59^c^	2.37 ± 0.55^g^	1.03 ± 0.12	55.14 ± 10.20^c^

Values are expressed as mean ± SD of five rats.

Yoghurt: Bulgaris, a brand of commercial yoghurt (Namyang, Korea).

FRe: fermented rice extracts.

BFRe: mixed formula consisting of yoghurt and the appropriate percentages (0.05, 0.1 and 1%) of FRe.

All solutions were administered as 10 mL/kg doses.

^a^ *P* < 0.01 and ^b^ *P* < 0.05 compared with the vehicle control by the LSD test.

^c^ *P* < 0.01 and ^d^ *P* < 0.05 compared with the loperamide control by the LSD test.

^e^ *P* < 0.01 and ^f^ *P* < 0.05 compared with the vehicle control by the MW test.

^g^ *P* < 0.01 and ^h^ *P* < 0.05 compared with the loperamide control by the MW test.

**Table 3:** Remnant fecal pellets in the colon of loperamide-induced constipated rats after oral treatment of the test agents.

Groups	Remnant fecal pellets in the colon	
	Numbers	Mean thicknesses (*μ*m)
Controls		
Vehicle	3.80 ± 0.84	2.69 ± 0.80
Loperamide	7.00 ± 1.00^a^	4.97 ± 0.13^e^
Single formula		
FRe 5 mg/kg	4.40 ± 0.89^c^	4.35 ± 0.40^e^
FRe 10 mg/kg	4.00 ± 0.71^c^	3.94 ± 0.06^eg^
Yoghurt	3.80 ± 0.84^c^	3.57 ± 0.36^g^
Mixed formula		
BFRe 0.05%	2.40 ± 1.67^bc^	3.32 ± 0.43^g^
BFRe 0.1%	1.60 ± 0.89^ac^	2.87 ± 0.83^g^
BFRe 1%	1.20 ± 0.45^ac^	2.18 ± 0.58^g^

Values are expressed as mean ± SD of five rats.

Yoghurt: Bulgaris, a brand of commercial yoghurt (Namyang, Korea).

FRe: fermented rice extracts.

BFRe: mixed formula consisting of yoghurt and the appropriate percentages (0.05, 0.1 and 1%) of FRe.

^a^ *P* < 0.01 and ^b^ *P* < 0.05 compared with the vehicle control by the LSD test.

^c^ *P* < 0.01 compared with the loperamide control by the LSD test.

^e^ *P* < 0.01 compared with the vehicle control by the MW test.

^g^ *P* < 0.01 compared with the loperamide control by the MW test.

**Table 4:** Gastrointestinal charcoal transit ratio in loperamide-induced constipated rats after oral treatment of the test agents.

Groups	Gastrointestinal motilities (during 30 min)		
	Total small intestine length (cm)	Length of charcoal meal transferred (cm)	Gastrointestinal charcoal transit ratio (%)
Controls			
Vehicle	119.60 ± 2.72	87.00 ± 6.81	72.68 ± 4.16
Loperamide	121.50 ± 2.50	60.80 ± 4.15^e^	50.07 ± 3.82^e^
Single formula			
FRe 5 mg/kg	119.70 ± 2.49	70.40 ± 4.97^eg^	58.86 ± 4.70^eg^
FRe 10 mg/kg	121.00 ± 3.32	73.10 ± 7.94^fg^	60.37 ± 5.71^fg^
Yoghurt	120.50 ± 1.87	76.00 ± 1.58^eg^	63.09 ± 1.88^eg^
Mixed formula			
BFRe 0.05%	122.30 ± 3.05	78.14 ± 12.61^h^	64.00 ± 10.99
BFRe 0.1%	119.60 ± 1.52	95.14 ± 7.43^g^	79.57 ± 6.49^g^
BFRe 1%	120.70 ± 4.84	104.70 ± 4.44^eg^	86.91 ± 6.18^eg^

Values are expressed as mean ± SD of five rats.

Yoghurt: Bulgaris, a brand of commercial yoghurt (Namyang, Korea).

FRe: fermented rice extracts.

BFRe: mixed formula consisting of yoghurt and the appropriate percentages (0.05, 0.1 and 1%) of FRe.

Charcoal transit ratio (%) = ((total small intestinal length − charcoal meal transit distance)/total small intestinal length) × 100.

^e^ *P* < 0.01 and ^f^ *P* < 0.05 compared with the vehicle control by the MW test.

^g^ *P* < 0.01 and ^h^ *P* < 0.05 compared with the loperamide control by the MW test.

**Table 5:** Histomorphometry of the colon and remnant fecal pellets in loperamide-induced constipated rats after oral treatment of the test agents.

Groups	Histomorphometry (at sacrifice)		
	Fecal pellet surface mucous thicknesses (*μ*m)	Mucous-producing cell numbers (cells/mm^2^)	Colon mucosa thicknesses (*μ*m)
Controls			
Vehicle	46.17 ± 5.02	673.80 ± 79.28	396.20 ± 63.85
Loperamide	15.01 ± 3.32^a^	150.80 ± 13.88^e^	224.76 ± 15.92^e^
Single formula			
FRe 5 mg/kg	22.40 ± 3.57^a^	298.20 ± 13.88^eg^	262.02 ± 17.97^eg^
FRe 10 mg/kg	40.24 ± 8.35^c^	395.20 ± 90.76^eg^	306.61 ± 17.79^eg^
Yoghurt	47.18 ± 4.42^c^	477.80 ± 32.51^eg^	309.61 ± 12.63^eg^
Mixed formula			
BFRe 0.05%	51.79 ± 6.90^c^	502.20 ± 49.00^eg^	344.76 ± 47.57^g^
BFRe 0.1%	87.50 ± 7.09^ac^	551.80 ± 45.18^fg^	369.91 ± 33.32^g^
BFRe 1%	107.53 ± 7.32^ac^	597.80 ± 57.01^g^	404.69 ± 12.10^g^

Values are expressed as mean ± SD of five rats.

Yoghurt: Bulgaris, a brand of commercial yoghurt (Namyang, Korea).

FRe: fermented rice extracts.

BFRe: mixed formula consisting of yoghurt and the appropriate percentages (0.05, 0.1 and 1%) of FRe.

^a^ *P* < 0.01 compared with the vehicle control by the LSD test.

^c^ *P* < 0.01 compared with the loperamide control by the LSD test.

^e^ *P* < 0.01 and ^f^ *P* < 0.05 compared with the vehicle control by the MW test.

^g^ *P* < 0.01 compared with the loperamide control by the MW test.
